# Scalable Fabrication of Organic Single-Crystalline Wafers for Reproducible TFT Arrays

**DOI:** 10.1038/s41598-019-50294-x

**Published:** 2019-11-04

**Authors:** Shohei Kumagai, Akifumi Yamamura, Tatsuyuki Makita, Junto Tsurumi, Ying Ying Lim, Takahiro Wakimoto, Nobuaki Isahaya, Han Nozawa, Kayoko Sato, Masato Mitani, Toshihiro Okamoto, Shun Watanabe, Jun Takeya

**Affiliations:** 1 Material Innovation Research Center (MIRC) and Department of Advanced Materials Science, Graduate School of Frontier Sciences, The University of Tokyo, 5-1-5 Kashiwanoha, Kashiwa, Chiba 277-8561 Japan; 2 International Center for Materials Nanoarchitectonics (WPI-MANA), National Institute for Materials Science (NIMS), 1-1 Namiki, Tsukuba, 305-0044 Japan; 3 Pi-Crystal Inc., 5-4-19 Kashiwanoha, Kashiwa, Chiba 277–882 Japan; 4 PRESTO, JST, 4-1-8 Honcho, Kawaguchi, Saitama 332-0012 Japan; 5 AIST-UTokyo Advanced Operando-Measurement Technology Open Innovation Laboratory (OPERANDO-OIL), National Institute of Advanced Industrial Science and Technology (AIST), 5-1-5 Kashiwanoha, Kashiwa, Chiba 277-8561 Japan

**Keywords:** Electronic devices, Organic molecules in materials science, Electronic properties and materials

## Abstract

Building on significant developments in materials science and printing technologies, organic semiconductors (OSCs) promise an ideal platform for the production of printed electronic circuits. However, whether their unique solution-processing capability can facilitate the reliable mass manufacture of integrated circuits with reasonable areal coverage, and to what extent mass production of solution-processed electronic devices would allow substantial reductions in manufacturing costs, remain controversial. In the present study, we successfully manufactured a 4-inch (*c.a.* 100 mm) organic single-crystalline wafer via a simple, one-shot printing technique, on which 1,600 organic transistors were integrated and characterized. Owing to their single-crystalline nature, we were able to verify remarkably high reliability and reproducibility, with mobilities up to 10 cm^2^ V^−1^ s^−1^, a near-zero turn-on voltage, and excellent on-off ratio of approximately 10^7^. This work provides a critical milestone in printed electronics, enabling industry-level manufacturing of OSC devices concomitantly with lowered manufacturing costs.

## Introduction

Organic semiconductors (OSCs) possess properties such as light weight, flexibility, and low-temperature processability that are ideal for the production of flexible, multi-functional electronic devices^1,2^. In particular, their unique solution-processability has highlighted their potential in printable electronics^3,4^. With the vast array of recent developments not only in printing technologies but also in materials science, the mass production of highly integrated OSC devices to meet looming IoT challenges is expected to be within our reach^5–7^. Although the electronic properties of organic devices have improved considerably, and extend far beyond those of amorphous silicon^8,9^, whether OSCs can promise reliable manufacturing of integrated circuits with reasonable areal coverage, and to what extent the mass production of solution-processed electronic devices can allow a substantial reduction of manufacturing costs, are still controversial issues. Concomitantly with the solution to manufacturing issues, highly reliable, reproducible transistors with high performance are a non-negotiable demand in electronic circuits.

A single crystal, which is a mono-domain solid in which a periodic lattice continues to the edges with no grain boundaries, is an ideal platform for highly integrated electronic circuits because of its uniformity. Particularly in the silicon electronics industry, the ultimate reliability is realized by the use of a single-crystal silicon wafer^10,11^. Recently, various groups have demonstrated the wafer-scale fabrication of ultra-thin single-crystal OSCs via a simple one-shot solution process^12–22^. The resulting excellent electronic properties, such as a field-effect mobility up to 10 cm^2^ V^−1^ s^−1^, allow high-speed switching operations at a few tenths of a MHz^16^.

Here, we verify a long-standing proof-of-concept in printed electronics: we successfully demonstrate the production of a 4-inch (*c*.*a*. 100 mm) organic single-crystalline wafer via a simple one-shot printing technique. With an active area of over 90 mm × 90 mm, 1,600 organic transistors composed of our benchmark OSC, 3,11-dinonyldinaphtho[2,3-*d*:2′,3′-*d*’]benzo[1,2-*b*:4,5-*b*’]dithiophene (C_9_–DNBDT–NW), are integrated. Excellent transistor properties with a field-effect mobility up to 12.5 cm^2^ V^−1^ s^−1^, a near-zero turn-on voltage, negligible hysteresis, and an on-off ratio of approximately 10 ^7^ are exhibited with remarkably high reliability and reproducibility.

## Results

### Wafer-scale printing: materials and manufacturing

**Figure 1:**
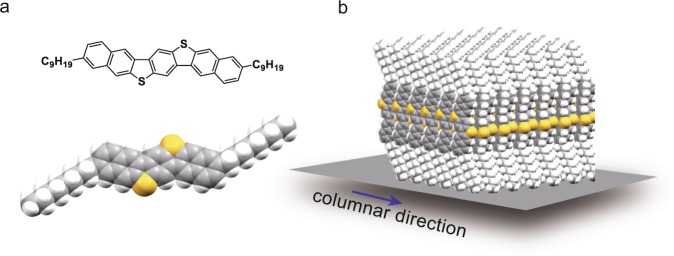
(**a**) Molecular structure of C_9_–DNBDT–NW. Upper: chemical structure; lower: space-filling model. (**b**) Schematic image of the structure of the molecular assembly. Both crystal growth and channel directions are along the columnar direction of the herringbone packing. Only one molecular layer is depicted in (**b**) for clarity.

**Figure 2:**
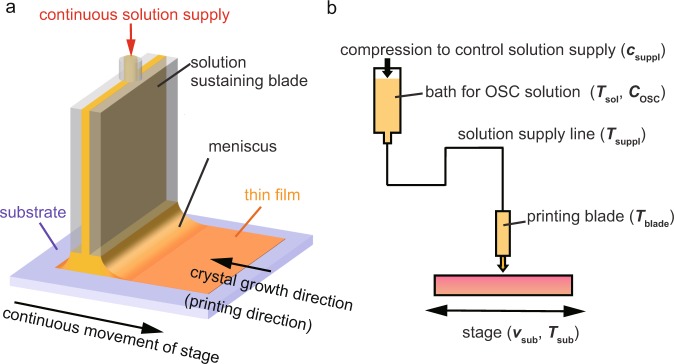
(**a**) Schematic illustration of continuous edge-casting method. OSC solution is continuously supplied from the top of the hollow solution-sustaining blade, during which the substrate, fixed on a stage, is unidirectionally moved. Organic thin-film crystals are grown in the direction opposite to that of stage movement. (**b**) Schematic illustration of the setup. To control the printing (crystal growth) conditions, the temperatures of the solution bath, solution supply lines, solution-sustaining blade and the stage can be monitored and controlled individually.

Our benchmark small-molecule OSC C_9_–DNBDT–NW (Fig. 1a,b)^23^ was employed to demonstrate large-area single-crystal formation by means of a printing technique. In our previous work, [2,3-*d*:2′,3′-*d*’]benzo[1,2-*b*:4,5-*b*’]dithiophene (DNBDT) analogs with different alkyl sidechains were used as representative organic semiconductors to form inch-size or wafer-scale single crystals by means of our sophisticated printing technique, termed continuous edge-casting^13,16^. In our experience, these alkyl-modified DNBDT derivatives are suitable for meniscus-driven printing owing to their adequate solubility and excellent two-dimensional crystallinity. Amongst them, C_9_–DNBDT–NW is likely the OSC with the best processability, owing to its high solubility. In order to manufacture large thin-film single crystals by means of continuous edge-casting, the solution-sustaining blade was modified to a width of 90 mm (Fig. 2a), which is suitable for 4-inch silicon wafers or any substrate with a side length of 100 mm. A key engineering task was to supply a homogeneous OSC solution with the desired concentration and temperature in order to reduce the concentration of undesirable nucleation centers. The apparatus, designed specifically for continuous edge-casting and constructed in-house, allows a continuous supply of OSC solution from a bath to the meniscus-holding blade, where the temperatures not only of the bath but also of all the lines that supply the solution are monitored and controlled precisely with an accuracy of ±1 °C. There is a uniaxially moving stage equipped to level the blade height and to shear the substrate underneath the blade.

Figure 2a schematically illustrates the growth of an organic single crystal by continuous edge-casting. Generally, for meniscus-guided crystal growth methods, solvent evaporation takes place predominantly at the vapor–liquid interface, resulting in solute supersaturation^16,19,21,22,24^. This initiates molecular aggregation at the surface of the solution and subsequent crystallization on the substrate. A self-assembled molecular nanosheet does not grow at the solid–liquid interface (the interface between the substrate and solution), but rather the vapor–liquid interface. This nanosheet is then laminated onto the substrate, which has been confirmed by *in situ* optical and X-ray observations during solution-shearing crystal growth^25^. Thus, balance between the solvent evaporation rate and the substrate movement speed is critical for controlling the uniformity and thickness of organic single crystals. Here, we summarize some critical parameters for obtaining homogeneous organic single-crystalline thin films with large areal coverage via continuous edge-casting. To be able to supply a homogeneous OSC solution with the desired concentration (*C*_OSC_) to the meniscus regime, temperatures must be monitored and controlled individually for the OSC solution bath (*T*_sol_), all solution supply lines (*T*_suppl_), and the solution-supplying blade (*T*_blade_), as shown in Fig. 2b. In addition, the rate of solution supply (*c*_suppl_), shearing velocity (*v*_sub_), and substrate temperature (*T*_sub_) directly determine the number of layers, from monolayer to multi-layer thickness. The specific parameters used for the deposition of trilayer C_9_–DNBDT–NW thin films via continuous edge-casting are summarized in Table 1. The parameters in Table 1 were determined through trial and error, and are classified into two groups. Parameters in group 1 (*T*_sol_, *T*_suppl_, and *T*_blade_) determine a continuous supply of OSC solution with the desired concentration onto a substrate. The process windows for these parameters are found to be relatively wide. For instance, a variation of 10 °C is acceptable for parameters *T*_sol_ and *T*_suppl_. When values outside the process window are used, the meniscus shape becomes unstable, which results in an uncontrollable deposition. For instance, a large temperature gradient between the blade and substrate would induce a Marangoni flow of the OSC solution at the air-liquid interface^26^. The second group of parameters (*C*_OSC_, *T*_sub_, *v*_sub_ and *c*_suppl_) controls the thickness of the molecular layers (monolayer, bilayer etc.). The thickness of the OSC thin films are predominantly influenced by the rate of solute precipitation and the solvent evaporation at the meniscus of a droplet retained at the blade *C*_OSC_ and *T*_sub_ are critical parameters which control the number of crystalline film layers, specifically from monolayer to multi-layer thickness. While homogeneous crystalline thin films can be obtained by optimizing the parameters in group 1, layered controlled thin films are obtained through a narrow process window. For instance, lowering *T*_sub_ by a few degrees results in a monolayer OSC film. On the other hand, parameters *v*_sub_ and *c*_suppl_ are important for balancing the rate of solvent evaporation and the volume of OSC solution retained at the blade^27,28^. With these parameters, C_9_–DNBDT–NW trilayer thin films were deposited onto a parylene (25 nm)/SiO_2_ (100 nm) hybrid gate dielectric. The thin parylene layer, which is deposited by chemical vapor deposition, is used to control the surface hydrophobicity and to minimize surface traps on the SiO_2_.

**Table 1:** Critical parameters in continuous edge-casting method for deposition of C_9_–DNBDT–NW trilayer.

*c*_suppl_ (*μ*l s^−1^)	*T*_sol_ (°C)	*C*_OSC_ (wt%)	*T*_suppl_ (°C)	*T*_blade_ (°C)	*v*_sub_ (*μ*m s^−1^)	*T*_sub_ (°C)
0.510	110	0.02	88	84	15	88

### Surface profile of wafer-scale organic single-crystal semiconductor

**Figure 3:**
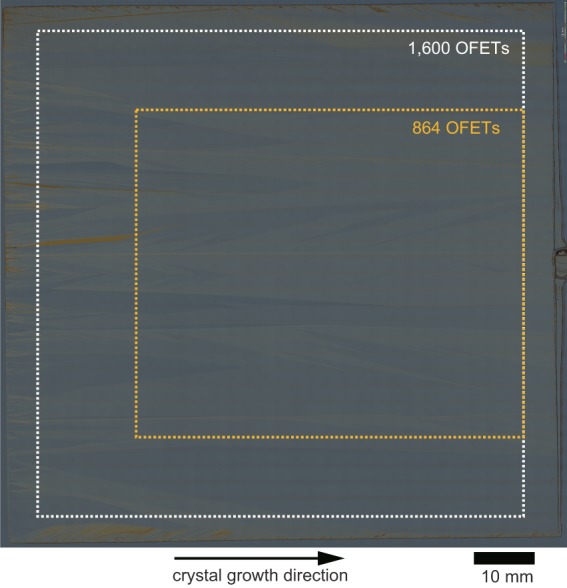
Confocal microscopy image of 90 mm by 90 mm C_9_–DNBDT–NW crystal on silicon wafer substrate. The direction of shearing, corresponding to the crystal growth direction, was from left to right. Scale bar: 10 mm. White and yellow dotted lines show the areas where the OFETs are located. From the crystal growth viewpoint, a mono-domain crystal without any defects is referred to as a “perfect crystal”. Yet a typical single crystal shows inherent lattice defects or impurities due to the third law of thermodynamics. On the other hand, a “poly-crystal” is defined as a multi-domain solid, where the orientation of microscopic crystals could be random with no preferential direction. The term “poly-crystal” is not used to define the crystalline thin films in this paper, because the orientation of our crystals has a preferential direction. Instead the term “single crystal” is used for our crystalline film. We foresee that the issue of misalignment could be overcome through an improvement of the printing apparatus and printing conditions.

Figure 3 shows a confocal microscope image of a 90 mm by 90 mm C_9_–DNBDT–NW thin film formed on the surface of the substrate. Here, we intentionally deposited trilayer C_9_–DNBDT–NW thin films in order to achieve sufficient robustness to withstand the subsequent lithography process^12,16,29–31^. Although some crystal facets were observed, uniform crystalline thin films were successfully formed across the entire substrate. Note that there are undesirably thick crystalline films present both at the entrance region, that is, the region where the meniscus-holding blade is initially positioned, and at the side edges of the substrate. These are unavoidable defects that are formed by any kind of meniscus-guided printing methodology. These issues can be solved in principle simply by widening the blade. Moreover, because the OSC solution bath is designed to contain a large excess of solution (specifically 20 ml for our apparatus), the areal coverage along the shearing direction can be easily increased by lengthening the substrate.

**Figure 4:**
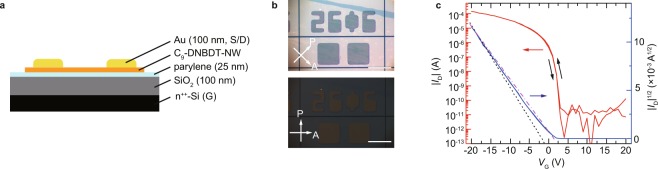
(**a**) Schematic illustration of device configuration. (**b**), Cross-polarized optical microscopy images of device. (**c**) Transfer characteristics of the device (*V*_D_ = −20 V). Black broken and magenta dot-dashed lines represent the fit and the slope for an ideal transistor, respectively. Channel length (*L*) and channel width (W) are 200 *μ*m and 500 *μ*m.

Based on the C_9_–DNBDT–NW film shown in Fig. 3, a 1600 (40 by 40 matrix) OFET array was fabricated in the top-contact bottom-gate geometry (schematically shown in Fig. 4a). The top-contact Au source (S) and drain (D) electrodes were prepared by means of photolithographic processing^12,16,29,30^. In addition, active OSC layers were electrically isolated by a laser etching technique to avoid cross-talk between neighboring OFETs (Fig. 4b). Thus, the mobility can be estimated accurately due to the absence of a fringe effect^32^. The channel width (*W*) and length (*L*) were 500 *μ*m and 200 *μ*m, respectively, so that *W*/*L* was 2.5. The capacitance of the parylene/SiO_2_ hybrid gate dielectric was measured as 26.3 nF cm^−2^. The formation of single-crystalline films was confirmed by cross-polarized optical microscopy images (Fig. 4b). When the crystal growth direction is parallel or perpendicular to the polarization angle, an almost completely black image is obtained, which indicates that the crystal axes are highly oriented. Although X-ray diffraction (XRD) and selected-area electron diffraction (SAED) measurements could be used to characterize the crystallinity of the thin films in this paper, the possible observation areas are limited with these equipment (approximately 300 *μ*m square for XRD and 100 nm square for SAED). As such a polarized microscope is used to identify the molecular layer thickness, as the optical intensity in the polarized microscope changes discretely with respect to molecular layer thickness. In the previous work, XRD and SAED measurements were performed concomitantly with cross-polarized optical microscopy measurements for DNBDT analogs with different alkyl sidechains^16^. Periodic diffraction patterns were observed from the measurements, which suggest that the resulting thin films are single crystals. The results are found to be consistent with those obtained from the polarized microscope measurements^16^.

Figure 4c shows the transistor characteristics of one of the fabricated OFETs in the array (*i* = 26, *j* = 06). The transfer characteristics in the saturation regime exhibit negligibly small hysteresis, a near-zero turn-on voltage, and a high on-off ratio of more than 10^7^, which is textbook-like behavior. The mobility was determined from the transconductance to be approximately 12.5 cm^2^ V^−1^ s^−1^ for the best OFET (statistical results will be discussed later). The threshold and turn-on voltages (*V*_th_ and *V*_on_, respectively) were −1.8 and +3 V, respectively. This mobility is as high as those previously reported by our group with DNBDT analogs^16,31^. The threshold and turn-on voltages (*V*_th_ and *V*_on_, respectively) were estimated to be −1.8 and +3 V, respectively. It should also be emphasized that the gate voltage dependence of the mobility determined from the transfer characteristics shows no “kink-down” behavior, which supports the validity of the estimated values^33,34^.

### Evaluation of single-crystal OFET array

**Figure 5:**
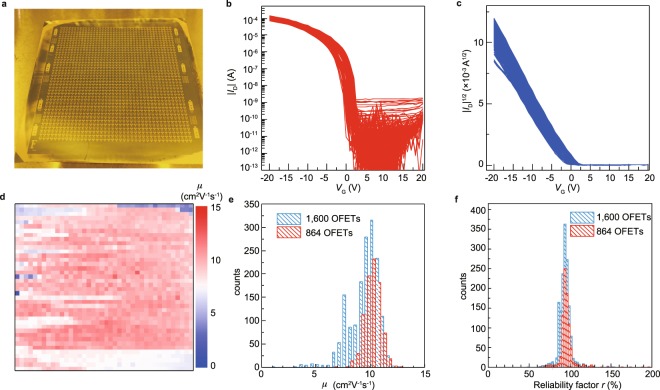
Evaluation of 1,600 (40 × 40 matrix) OFETs. (**a**) Photograph of OFET array. (**b,c**) Transfer curves (*V*_D_ = −20 V) obtained for 864 OFETs at the center of wafer (27 × 32 matrix). (**d**) Mobility map for all 1,600 OFETs. (**e**) Statistics for measured mobility. (**f**) Statistics for measured reliability factor *r*.

Although 1,600 (40 × 40) OFETs were fabricated on the 90 mm × 90 mm film (Fig. 5a), we evaluated 864 (27 × 32) in the area outlined in orange in Fig. 3. This is because the film was undesirably thick within ~25 mm from the entrance region and within ~20 mm from the side edges of the substrate due to imperfect nucleation. However, it is noteworthy that the extracted OFETs came from a continuous large single-crystalline domain at the center of the C_9_–DNBDT–NW thin film (approximately 64 mm × 54 mm), which still has dimensions of a few tenth of millimeters. Figure 5b,c show representative transfer curves. Almost identical transfer characteristics were obtained for all OFETs.

Next, the statistical results for the case of 864 and 1,600 OFETs were evaluated. The mobility (*μ*) distribution is shown in Fig. 5d,e. For the central 864 OFETs, the mobility is uniformly high, with an average value of 10.1 cm^2^ V^−1^ s^−1^ and a standard deviation of 0.73 cm^2^ V^−1^ s^−1^. For the entire 1,600 OFETs, the average and standard deviation are 9.5 and 1.35 cm^2^ V^−1^ s^−1^, respectively. These values are comparable to those reported for analogous DNBDT semiconductor layers^16,31^. The slightly lower mobility values in the present study might be due to the uncontrolled crystal thickness. At the bottom region (approximately 30 × 5 elements) of Fig. 5d, another large single-crystalline domain can be recognized as whitish pixels (*μ* ~ 7.5 cm^2^ V^−1^ s^−1^). 162 OFETs are contained in this domain, and exhibit an average mobility of 7.71 cm^2^ V^−1^ s^−1^ with a standard deviation of 0.32 cm^2^ V^−1^ s^−1^. The relatively low mobility in this domain is likely to be mainly due to its crystallographic axis being different from that in the central (higher *μ*) domain, which is not conducive to the best hole transport. Currently, mobility evaluation in OFETs is the subject of active debate, so the reliability factor proposed by Podzorov *et al*. was calculated^35^. The reliability factor was 81.8% (standard deviation 9.1%) for the central 864 OFETs, and 94.0% (standard deviation 9.1%) for the entire 1,600 OFETs (Fig. 5f). These mobility values are comparable to (or rather better than) that for a single-crystal transistor array based on C_8_-BTBT recently reported by Chan *et al*.^21^.

## Discussion

Here, we again summarize our findings. Continuous edge-casting, which is a simple, one-shot meniscus-guided printing technique, allowed production of a 4-inch (*c*.*a*. 100 mm) organic single-crystalline wafer. All 1,600 OFETs fabricated on the surface of the wafer operated with a reasonably high field-effect mobility of 9.5 cm^2^ V^−1^ s^−1^ (standard deviation 1.35 cm^2^ V^−1^ s^−1^). Importantly, even though the edges of the semiconductor film have unavoidable crystal misalignments, likely due to imperfect nucleation, these issues can be solved in principle. Indeed, OFETs located at the center of the array, which are free from imperfection due to misalignments, showed better OFET performance and a narrower mobility distribution. Therefore, further technical improvements to produce more uniform and larger single crystals promise to deliver large-area printed electronics with very high carrier mobility and reproducibility, leading to high-end OFET-based devices in the near future.

Lastly, we discuss how the present continuous edge-casting would reduce the manufacturing costs. Firstly, printing crystalline films over the whole substrate would be advantageous from a time-cost consideration. Generally, meniscus-guided coating methods adopt a unidirectional printing and the total deposition time is determined predominantly by the product of the shearing speed (*v*_sub_) and the shearing distance (*l*_sub_). Thus, widening the blade width and substrate width (*w*_sub_) enlarges the coverage of the crystalline film. This is in contrast to printing methods which require a wide area scan (*e*.*g*. inkjet printing^3,36^), where the factor *l*_sub_ × *w*_sub_ would determine the total deposition time. As such large area scalability could be expected in meniscus-guided methods. Comparing meniscus-guided coating methods to area selective coating methods (*e*.*g*. inkjet printing^3,36^, stamp printing^37^), the former requires an etching process to form active channel islands which results in the wastage of semiconductor crystals. In this case, an active crystalline channel is formed isolated to minimize the cross-talk between neighboring transistors. However, this would not have a significant effect on the cost reduction for high density integration, as the active channel area is increased more than the etching area. Furthermore, as the single crystalline films are composed of only a few molecular layers, the consumption of raw materials is minimal. For instance, approximately 100 *μ*g of C_9_–DNBDT–NW was used for the present areal coverage of 100 cm^2^, which corresponds to approximately 60 ng per each transistor. Although further studies are needed, an effective cost reduction would be achievable by increasing the degree of integration. The apparatus used in this work requires a constant heating of the organic semiconductor solution, which could result in an additional cost. Since our benchmarked C_9_–DNBDT–NW does not have an extremely high solubility at room temperature, a relatively high temperature process is necessary. However, we do not think that heating the entire solution-supplying system would be disadvantageous. Generally, moderate heating in printing processes, particularly those involving organic solvents is likely to accelerate the solvent evaporation process, resulting in significant time saving. Thus, substantial reductions in manufacturing costs could be achieved by taking into account the time-saving and cost-saving benefits involved.

## Methods

### Materials and OFET array fabrication

C_9_–DNBDT–NW was synthesized and purified in-house. OFETs were prepared on a silicon wafer substrate with surface modification by parylene, diX-SR (KISCO Ltd.). A 6-inch, highly-doped n^++^ silicon wafer with a 100 nm-thick thermally oxidized SiO_2_ layer was encapsulated by 25 nm-thick diX-SR, where the capacitance per unit area (*C*_i_) of the SiO_2_ and diX-SR bilayer gate dielectric was measured to be 26.3 nF cm^−2^. A 0.02 wt% solution of C_9_–DNBDT–NW in 3-chlorothiophene was prepared at 105 °C. Before forming C_9_–DNBDT–NW films, the substrate was shaped into a 100 mm × 100 mm square. The continuous edge-casting method using a solution-supplying blade with a width of 90 mm was applied to form a large-area single crystal of C_9_–DNBDT–NW, where the blade was fixed and the stage holding the substrate was moved at 15 *μ*m s^−1^ in the direction opposite to that of crystal growth. The crystal was then placed in a vacuum oven at 80 °C for 10 hours to thoroughly remove the residual solvent. Source and drain electrodes were prepared by multiple photolithographic processes, where OSCoR4001 (Orthogonal Inc.) and AURUM S-50790 (Kanto Chemical Co. Inc.) were employed as the photoresist and gold etchant, respectively, forming patterned, top-contact 100 nm-thick Au layers. An OFET array with a 40 × 40 matrix was prepared. The individual OFETs were edge etched using a conventional yttrium-aluminum-garnet laser. Each OFET had a channel width (*W*) of 500 *μ*m and a length (*L*) of 200 *μ*m (*W*/*L* = 2.5).

### Electrical measurements

All electrical measurements were conducted with a semiconductor parameter analyzer (Keithley 4200-SCS) in conjunction with a semi-automatic probe station (HiSOL HSP-150) under dark and ambient conditions. The mobility, *μ*, in the saturation regime was determined from the transfer characteristics using:

$${I}_{{\rm{D}}}=\frac{\mu W{C}_{{\rm{i}}}}{2L}{({V}_{{\rm{G}}}-{V}_{{\rm{th}}})}^{2}$$

where *I*_D_, *L*, *W*, *C*_i_, *V*_G_, *V*_th_, and *V*_D_ are the drain current, channel length, channel width, capacitance per unit area, gate voltage, threshold voltage, and drain voltage, respectively. The values of *C*_i_ were determined from capacitance–voltage measurements at a frequency of 5 kHz.

To assess the validity of the estimated mobility, the reliability factor^35^, *r*, in the saturation regime was calculated using the following equation:

$$r={(\frac{\sqrt{{|{I}_{{\rm{D}}}|}^{{\rm{\max }}}}-\sqrt{|{I}_{{\rm{D}}}^{0}|}}{{|{V}_{{\rm{G}}}|}^{{\rm{\max }}}})}^{2}/(\frac{W{C}_{{\rm{i}}}}{2L}\mu )$$

where |*I*_D_|^max^ is the maximum drain current at the maximum gate voltage |*V*_G_|^max^ and ${I}_{{\rm{D}}}^{0}$ is the drain current at *V*_G_ = 0.

## Data Availability

The data that support the plots in this paper and the other findings of this study are available from the corresponding author (Shohei Kumagai; s-kumagai@edu.k.u-tokyo.ac.jp) upon request.
